# Abnormal Spatial and Temporal Overlap of Time-Varying Brain Functional Networks in Patients with Schizophrenia

**DOI:** 10.3390/brainsci14010040

**Published:** 2023-12-31

**Authors:** Jie Xiang, Yumeng Sun, Xubin Wu, Yuxiang Guo, Jiayue Xue, Yan Niu, Xiaohong Cui

**Affiliations:** 1 College of Computer Science and Technology, Taiyuan University of Technology, Taiyuan 030024, China; xiangjie@tyut.edu.cn (J.X.); m15735216932@163.com (Y.S.); wuxubin0066@link.tyut.edu.cn (X.W.); xjayoo@163.com (J.X.); niuyan@tyut.edu.cn (Y.N.); 2 School of Software, Taiyuan University of Technology, Taiyuan 030024, China; sxtygyx@163.com

**Keywords:** time-varying functional connectivity, candidate hubs, active hubs, spatial overlap, temporal overlap

## Abstract

Schizophrenia (SZ) is a complex psychiatric disorder with unclear etiology and pathological features. Neuroscientists are increasingly proposing that schizophrenia is an abnormality in the dynamic organization of brain networks. Previous studies have found that the dynamic brain networks of people with SZ are abnormal in both space and time. However, little is known about the interactions and overlaps between hubs of the brain underlying spatiotemporal dynamics. In this study, we aimed to investigate different patterns of spatial and temporal overlap of hubs between SZ patients and healthy individuals. Specifically, we obtained resting-state functional magnetic resonance imaging data from the public dataset for 43 SZ patients and 49 healthy individuals. We derived a representation of time-varying functional connectivity using the Jackknife Correlation (JC) method. We employed the Betweenness Centrality (BC) method to identify the hubs of the brain’s functional connectivity network. We then applied measures of temporal overlap, spatial overlap, and hierarchical clustering to investigate differences in the organization of brain hubs between SZ patients and healthy controls. Our findings suggest significant differences between SZ patients and healthy controls at the whole-brain and subnetwork levels. Furthermore, spatial overlap and hierarchical clustering analysis showed that quasi-periodic patterns were disrupted in SZ patients. Analyses of temporal overlap revealed abnormal pairwise engagement preferences in the hubs of SZ patients. These results provide new insights into the dynamic characteristics of the network organization of the SZ brain.

## 1. Introduction

Schizophrenia is a serious, chronic mental illness that often develops in early adulthood [1]. Hallucinations, delusions, speech and behavioral disturbances, and mood disorders are the hallmark symptoms of this psychiatric disorder [2]. Neuroimaging studies have facilitated extensive research into schizophrenia, and complex network analysis methods are frequently utilized in this field. Research has shown that schizophrenia is a complex psychiatric disorder characterized by abnormalities in whole-brain connectivity and is associated with abnormal functional coordination between multiple brain regions.

With the advancement of neuroscience tools, various neuroimaging methods, such as electroencephalography (EEG)/intracranial electroencephalography (iEEG), functional magnetic resonance imaging (fMRI), magnetoencephalography (MEG), and event-related potentials (ERPs), have been employed to investigate brain function [3,4,5,6]. Notably, functional magnetic resonance imaging (fMRI) stands out due to its exceptional spatial resolution, enabling precise anatomical localization and visualization of deep brain structures [7]. This method offers comprehensive whole-brain coverage, facilitating the exploration of functional connectivity and network-level brain function, distinguishing it from other techniques that may have limitations in capturing activity across the entire brain simultaneously [8]. Furthermore, fMRI’s capacity to measure blood flow changes provides insights into hemodynamic responses associated with neural activity, aiding in the identification of anatomical correlates of cognitive function [9]. Resting-state functional magnetic resonance imaging (rs-fMRI) has become an essential tool for exploring the functional networks of the human brain. Traditional methods have assumed stable functional connectivity throughout scans when constructing brain networks. However, recent studies have shown that the human brain is dynamic, displaying scale-free fluctuations in space and time [10]. As such, dynamic network analysis methods have been increasingly adopted to better detect fluctuations in functional brain connectivity [11,12]. For instance, Cui et al. utilized dynamic functional connectivity to capture changes in network topology and cognitive behavior over time, providing a better understanding of the time-varying characteristics of brain networks [13]. Similarly, Braun et al. combined pharmacological interventions with novel techniques in dynamic network neuroscience to study alterations in the dynamic remodeling of brain networks associated with genetic risks for schizophrenia [14]. Meanwhile, Zhu et al. used sliding windows and Pearson correlation to construct dynamic brain networks to study functional connectivity abnormalities and network disruptions in the whole brain [15]. Additionally, Gifford et al. introduced a new method for measuring large-scale dynamic brain organization called dynamic modular organization. They used it to examine intergroup differences in dynamic community structure in SZ [16]. These studies collectively underscore the growing recognition that schizophrenia is a disorder caused by abnormalities in the dynamic organization of brain networks [17,18,19].

Previous studies have focused on changes in functional connectivity within brain networks and changes in specific brain communities. However, graph theory-based network analyses have shown that the presence of hubs represents a vital feature of the organization of large-scale brain networks. As highly connected regions, hubs integrate and distribute information throughout the brain and facilitate effective communication between different brain regions. Despite this, previous investigations have neglected to examine hubs’ dynamic reorganization and spatiotemporal patterns. Our study, therefore, aims to fill this gap by examining the dynamic spatiotemporal organization patterns of hubs in patients diagnosed with schizophrenia through temporal overlap, spatial overlap, and hierarchical clustering [20,21].

Here, we aimed to investigate abnormal changes in the dynamic spatiotemporal organization of brain hubs in SZ patients at rest, thus providing a new characterization of the dynamic network in SZ patients. Specifically, we investigate the spatiotemporal organization of hubs in dynamic brain networks based on a time-varying approach to functional connectivity. A time-varying network is established using the Jackknife Correlation (JC) method to obtain the dynamic functional connectivity of the network, and the Betweenness Centrality (BC) method is used to identify the hubs in the brain. Temporal overlap, spatial overlap, and hierarchical clustering are then used to quantify fluctuations in the brain hubs.

## 2. Materials and Methods

### 2.1. Participants

The data were selected from the UCLA Consortium for Neuropsychiatric Phenomics LA5c Study project, which was approved by a UCLA Institutional Review Board. These data were obtained through a public database, open fMRI (https://openfmri.org/dataset/ds000030/, accessed on 1 September 2020). Our study included 92 participants, including 43 people with schizophrenia (SZ) and 49 normal controls (NC). There were no significant differences in age and sex between NC and SZ (*t*-test). It is worth noting that 43 SZ patients out of 92 subjects participated in the SAPS assessment. The SAPS (Scale for the Assessment of Positive Symptoms) is a clinical rating scale used to assess positive symptoms of schizophrenia, including hallucinations, delusions, and disorganized thinking. Specific demographic characteristics are shown in Table 1.

### 2.2. Imaging Acquisition and Preprocessing

All subjects underwent MRI scans with 32-channel head coils on a 3-T scanner at the University of Electronic Science and Technology of China. Resting-state fMRI data were collected using a series of T2*-weighted echo plane imaging (EPI). The parameters are as follows: Repeat Time (TR) = 2 s, Echo Time (TE) = 30 ms, Slice Thickness = 4 mm, Slices = 34, Flip Angle = 90°, Field of View (FOV) = 192 mm, Matrix = 64 × 64. The resting-state fMRI scan lasted a total of 304 s. For the structure scan, a T1-weighted high-resolution anatomical scan was performed using the following parameters: slice thickness = 1 mm, slices = 176, repeat time (TR) = 1.9 s, echo time (TE) = 2.26 ms, matrix = 256 × 256, field of view (FOV) = 250 mm.

Data preprocessing was carried out using the DPABI toolbox (a toolbox for Data Processing & Analysis for Brain Imaging, V2.1, http://rfmri.org/dpabi, accessed on 30 July 2023), the first 10 volumes of signals were discarded, and data for the remaining 142 time points were preprocessed as follows: (1) Slice timing correction. (2) Realignment. (3) Normalization: Image space normalized to the Montreal Neurological Institute (MNI) head anatomy template and resampled with 3 × 3 × 3 mm^3^ voxels [22]. (4) Filtering: bandpass filtering (0.01 ≤ f ≤ 0.1 Hz) was performed on the image. (5) Smoothing: the images were spatially smoothed using a Gaussian filter with a full-width at half-maximum (FWHM) of 6 mm [23]. (6) Remove the covariates and divide the brain into 90 regions using the Automated Anatomical Marker Template (AAL) [24], with each voxel extracting the residual time series.

Based on the time series extracted from the AAL template (Detailed information is provided in Table A1), we divided brain regions into 5 functional networks [25]: somatosensory/motor and auditory network (SMN), visual network (VN), attention network (AN), default mode network (DMN), and limbic/paralimbic and subcortical network (LSN).

### 2.3. Calculation of Time-Varying Functional Connectivity (TVC)

We used the Jackknife Correlation (JC) method to evaluate functional connectivity over time [26]. The JC of two BOLD time series x and y at time point t can be expressed as

(1) $$JC_{t}=-\left(\frac{\Sigma_{t_{i}}^{t_{total}}\left(x_{t_{i}}-\bar{x_{t}}\right)\left(y_{t_{i}}-\bar{y_{t}}\right)}{\Sigma_{t_{i}}^{t_{total}}{\left(x_{t_{i}}-\bar{x_{t}}\right)}^{2}\Sigma_{t_{i}}^{t_{total}}{\left(y_{t_{i}}-\bar{y_{t}}\right)}^{2}}\right),\;t_{i}\neq t$$

where $t_{total}$ is the total number of time points. $\bar{x_{t}}$ and $\bar{y_{t}}$ are the expected values excluding the data at time point t:

(2) $$\begin{array}{c} \bar{x_{t}}=\frac{1}{t_{total}-1}\sum_{t_{i}}^{t_{total}}x_{t_{i}},\;t_{i}\neq t \end{array}$$

(3) $$\begin{array}{c} \bar{y_{t}}=\frac{1}{t_{total}-1}\sum_{t_{i}}^{t_{total}}y_{t_{i}},\;t_{i}\neq t \end{array}$$

Notably, previous studies have shown that the JC method performs better in tracking covariance time changes than other methods. Here, each JC value is normalized, and the standardized JC method is unaffected by the underlying static properties. The JC method estimates paired functional brain connections at each time point. This information can be used to investigate the dynamic characteristics of a given network and the interactions between different nodes within it.

### 2.4. Calculation of Betweenness Centrality at Multilevel

In order to identify candidate hubs and active hubs in the network, it is necessary to calculate the Betweenness Centrality (BC) [27,28] value for each node using a graph measure. This metric is a basis for determining whether a given node is a hub. BC is a type of network centrality metric that quantifies the influence of a particular node in connecting other nodes within the network. Nodes with high BC values are often called “hubs” or “high centers”. These nodes are critical in inter-node communication, facilitating information transfer between other nodes. The normalized BC of node $n_{i}$ is defined as

(4) $${BC}_{n_{i}}=\frac{2}{\left(n_{total}-1\right)\left(n_{total}-2\right)}\sum_{\begin{array}{c} n_{h},{\;n}_{j}\in N\\ n_{h}\neq n_{j},{\;n}_{h}\neq n_{i},\;n_{j}\neq n_{i} \end{array}}\frac{\rho_{n_{h}n_{j}}\left(n_{i}\right)}{\rho_{n_{h}n_{j}}}$$

where $n_{total}$ is the total number of all nodes, N is the set containing all nodes, $\rho_{n_{h}n_{j}}$ is the number of shortest paths between $n_{h}$ and $n_{j}$, and $\rho_{n_{h}n_{j}}\left(n_{i}\right)$ is the number of shortest paths between node $n_{h}$ and node $n_{j}$ that pass through node $n_{i}$.

Meanwhile, we calculated the time average of BC for node $n_{i}$ as

(5) $${BC\_avg}_{n_{i}}=\frac{1}{t_{total}}\Sigma_{t=1}^{t_{total}}{BC}_{n_{i}}\left(t\right)$$

After calculating the BC values at the node level, to further analyze our results, we studied the BC values at the global level and the resting-state network (RSN) level. By examining the BC values at different levels, we can better understand the network structure. Precisely, the BC values at each level were calculated using the following formulas:

Global BC: The sum of the BC values of all nodes divided by the total number of nodes in the network. Global BC is defined as

(6) $${BC\_avg}_{global}=\frac{1}{n_{total}}\Sigma_{n_{i}=1}^{n_{total}}{BC\_avg}_{n_{i}}$$

RSN BC: The average BC value of nodes belonging to a given RSN in the network. BC for ${RSN}_{j}$ is defined as

(7) $${BC\_avg}_{{RSN}_{j}}=\frac{1}{n_{{RSN}_{j}}}\sum_{n_{i}\in N_{{RSN}_{j}}}{BC\_avg}_{n_{i}}$$

where $n_{{RSN}_{j}}$ is the total number of nodes in ${RSN}_{j}$, $n_{i}$ is the set of all nodes contained in ${RSN}_{j}$, $N_{{RSN}_{j}}$ is the set of nodes in ${RSN}_{j}$.

### 2.5. Identification of Candidate Hubs and Active Hubs

A node is considered a candidate hub if it exhibits a higher time-averaged Betweenness Centrality (BC) value than other nodes. To identify candidate hubs, a threshold value of one standard deviation above the mean BC value is used, as represented by ${TH}_{1}$ [29]. Nodes with BC values above this threshold are selected as candidate hubs, allowing for targeted analysis of high-impact nodes within the network.

(8) $${TH}_{1}=\frac{1}{n_{total}}\Sigma_{n_{i}=1}^{n_{total}}{BC\_avg}_{n_{i}}+\sqrt{\frac{{\Sigma_{n_{i}=1}^{n_{total}}{(BC\_avg}_{n_{i}}-\frac{1}{n_{total}}\Sigma_{n_{i}=1}^{n_{total}}{BC\_avg}_{n_{i}})}^{2}}{n_{total}-1}}$$

Our study recognizes that hubs are not static and can change over time. Thus, we examined candidate hubs from a time-varying perspective. Among the candidate hubs, we define those with larger time-varying BC values as active hubs. In other words, we focused on active hubs with time-varying properties. Active hubs are a subset of candidate hubs with larger BC values at specific time points. A separate threshold is applied to the time-varying BC values of candidate hubs to identify active hubs, as represented by ${TH}_{2}$ [29]. Specifically, an active hub is defined as a candidate hub whose BC value exceeds one standard deviation of its average BC value. By identifying and analyzing active hubs, researchers can gain insight into each node’s dynamic role in the network’s overall connectivity structure over time.

(9) $${TH}_{2}={BC\_avg}_{n_{i}}+\sqrt{\frac{{\Sigma_{t=1}^{t_{total}}({BC}_{n_{i}}\left(t\right)-{BC\_avg}_{n_{i}})}^{2}}{t_{total}-1}}$$

### 2.6. Subnetwork Distribution of Active Hubs

In this section, our study aimed to investigate the properties of active hubs in different brain subnetworks. In other words, the distribution of active hubs in different subnetworks was studied. The evidence suggests that the number of active hubs fluctuates over time and exhibits different subnetwork distributions for different subnetworks [30]. To investigate whether the spatial configuration of active hubs differed within any particular subnetwork, we calculated the relative number of active hubs in each subnetwork for all subjects at all points in time. This approach allowed us to determine whether any particular subnetwork had more active hubs than others. The subnetwork distribution of active hubs (SND) belonging to a particular ${RSN}_{j}$ in the network is defined as:

(10) $${SND}_{{RSN}_{j}}=\frac{\sum_{n_{j}\in C\cap n_{j}\in N_{{RSN}_{j}},\;t\in T}A_{n_{j}}(t)}{\sum_{n_{i}\in C,\;t\in T}A_{n_{i}}(t)}$$

where $A_{n_{i}}(t)=1$ if node $n_{i}$ is an active hub at time t. C is the set of candidate hubs. $N_{{RSN}_{j}}$ is the set of nodes in ${RSN}_{j}$. T is the set of all time points.

### 2.7. Spatial Overlap and Temporal Overlap of Active Hubs

#### 2.7.1. Spatial Overlap of Active Hubs

Spatial overlap (SO) measures the spatial patterns of brain activity and provides valuable insight into the quasi-periodic nature of brain function. Specifically, SO involves the identification of reliable patterns of brain activity that are repeated over time. Mathematically, the degree of SO at time points $t_{i}$ and $t_{j}$ is calculated as the ratio of the intersection of active hubs at the two time points divided by the union of active hubs at those two time points. In this way, the spatial overlap between active hubs at different time points can be precisely quantified, providing insight into brain connectivity dynamics. The SO at time points $t_{i}$ and $t_{j}$ is expressed as:

(11) $$SO(t_{i},t_{j})=\frac{\left|S_{t_{i}}\cap S_{t_{j}}\right|}{\left|S_{t_{i}}\cup S_{t_{j}}\right|}$$

where $S_{t_{i}}$ is the set of active hubs at time point $t_{i}$, and $S_{t_{j}}$ is the set of active hubs at time point $t_{j}$.

#### 2.7.2. Hierarchical Clustering of Active Hubs

To explore the quasi-periodic patterns of brain space configurations, we utilized a top-down hierarchical clustering algorithm [31] to cluster these patterns in a rational way. In this approach, each pattern represents the set of active hubs at each time point, and we used spatial overlap between patterns as a similarity metric. We used the average linkage method to calculate the cluster-to-cluster distance. The results following hierarchical clustering provide insight into the quasi-periodic patterns of the spatial configuration of the brain in SZ and NC.

#### 2.7.3. Temporal Overlap of Active Hubs

Temporal overlap (TO) measures the interaction of the brain hubs’ activity, providing insight into the frequency patterns at which pairs of active hubs co-occur. In other words, TO represents the proportion of time during which paired active hubs are both active. By examining the degree of TO between hubs, we can gain essential insights into the pairwise participation of these hubs. Mathematically, the TO between compute nodes $n_{i}$ and $n_{j}$ can be expressed as the proportion of time during which both nodes are active hubs. This allows for the precise quantification of the degree of temporal overlap between candidate hubs, which can be used to identify hubs that exhibit particularly strong or consistent patterns of pairwise activity. By analyzing the temporal overlap of active hubs across different time points, researchers can gain insight into the temporal dynamics of activity within the brain network under study. The time overlap of compute nodes $n_{i}$ and $n_{j}$ is expressed as:

(12) $$TO(n_{i},n_{j})=\frac{\left|S_{n_{i}}\cap S_{n_{j}}\right|}{t_{total}}$$

where $S_{n_{i}}$ is the set of time points where node $n_{i}$ is an active hub, and $S_{n_{j}}$ is the set of time points where node $n_{j}$ is an active hub and $t_{total}$ is the number of total time points.

### 2.8. Statistical Analysis

**Figure 1:**
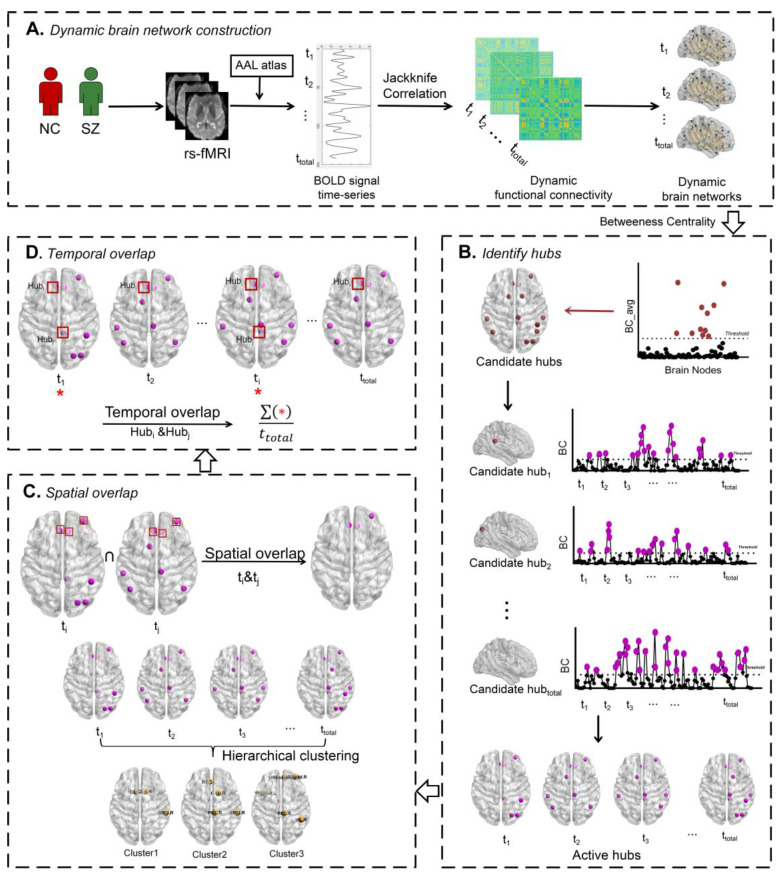
Overview of the schematic diagram of the analysis strategy. (**A**) Firstly, the standard brain contour present in the AAL template was divided into 90 individual brain regions. Each of these regions represented a node within the network under study. The JC method was then employed to estimate functional connectivity at each time point. (**B**) Next, the BC method was used to identify candidate hubs within the network that required tracking. The activity of these candidate hubs was observed over time to gain further insight into their interactions with other nodes within the network. Significant differences between SZ and NC were analyzed by examining (**C**) the spatial overlap, (**D**) temporal overlap, and hierarchical clustering of hubs.

An independent sample *t*-test was used for statistical tests to quantify the differences between NC and SZ. For each metric, we averaged the metrics obtained from the network. The Benjamini and Hochberg error discovery rate (BH_FDR) method was used to calibrate all the results, and the threshold value for the significant difference was set to <0.05. The pipeline of the analysis strategy for this study is displayed in Figure 1.

## 3. Results

### 3.1. Group Comparisons on BC Values

**Figure 2:**
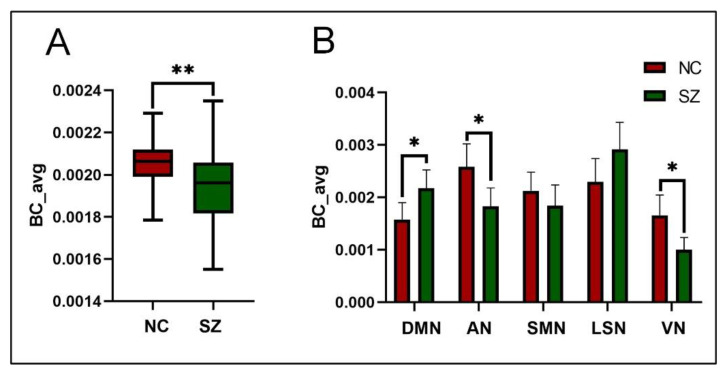
(**A**) Differences in BC at the whole-brain level. Asterisks represent group differences; ** denotes p < 0.01. (**B**) Group differences in BC at the RSN level. Asterisks represent the difference between groups; * denotes p < 0.05.

This study aimed to identify hubs by using BC values and assess the differences in BC between the two groups of subjects. We analyzed these differences at different levels, which included the whole brain and the resting state network (RSN). At the whole-brain level, our results showed that the BC values of SZ patients were significantly lower than those of NC subjects (*p* < 0.01), as shown in Figure 2A. At the RSN level, when using FDR correction to control for multiple comparisons, we found significantly higher BC in the default mode network (DMN) (*p* (FDR) < 0.05) and significantly lower BC in the attentional network (AN) (*p* (FDR) < 0.05) and visual network (VN) (*p* (FDR) < 0.05) in SZ patients compared to the NC group (Figure 2B).

### 3.2. Candidate Hubs

**Figure 3:**
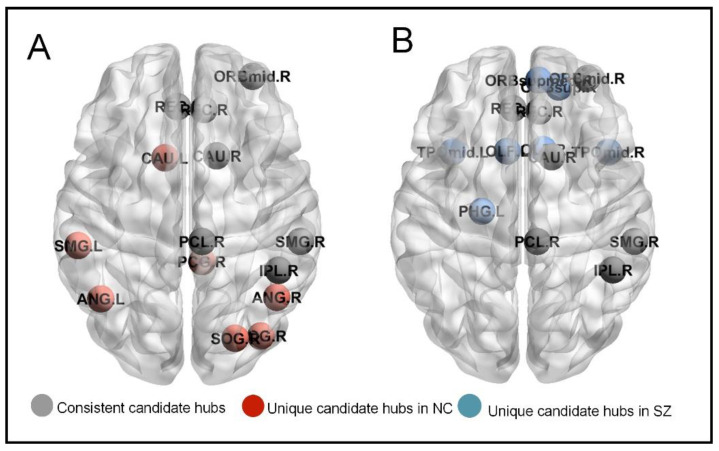
Distribution of candidate hubs in two groups: (**A**) NC, (**B**) SZ.

Our study also identified candidate hubs associated with larger BC values, which are shown in Figure 3. We found significant differences in the anatomical positioning of candidate hubs between SZ patients and NC subjects. In the NC group, we identified a total of 14 candidate hubs, which were distributed among DMN, AN, SMN, LSN, and VN. Specifically, there were three candidate hubs in DMN, four in AN, three in SMN, two in LSN, and two in VN. In contrast, in the SZ group, we identified 14 candidate hubs. These candidate hubs consisted of five in DMN, two in AN, two in SMN, and five in LSN. Of note, seven candidate nodes were consistent between the two groups.

### 3.3. Distribution of Active Hubs

**Figure 4:**
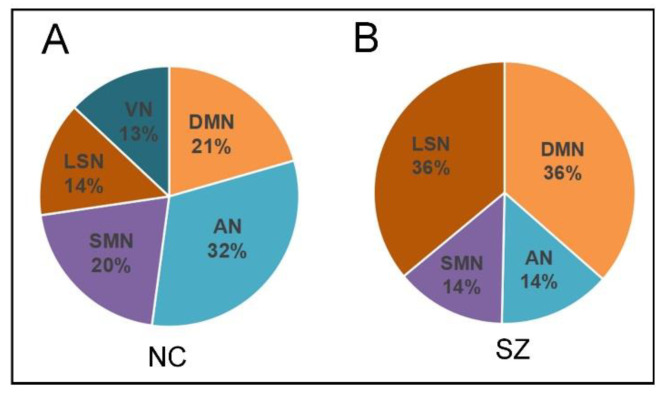
(**A**) Subnetwork distribution of active hubs in NC. (**B**) Subnetwork distribution of active hubs in SZ patients. The distribution of subnetwork members of active hubs at all time points under the average of all subjects is shown in the form of a pie chart.

We investigated the active hubs from the perspective of subnetwork distribution. It has been shown that the number of hubs fluctuates over time and varies for the distribution of different subnetworks in the brain. To explore whether the spatial configuration of active hubs differed in any system, we calculated the relative number of active hubs in each subnetwork at all time points for all subjects. The results are displayed in Figure 4. We can see that the relative proportion of active hubs in each subnetwork differs between NC and SZ groups. The most significant proportion of active hubs for the NC group is found in the AN, followed by DMN and SMN. The LSN and VN have the lowest proportion of active hubs. For the SZ group, the most significant proportion of active hubs is found in the DMN and LSN, followed by AN and SMN. The VN has the lowest proportion of active hubs in this group.

### 3.4. Spatial Overlap of Active Hubs

**Figure 5:**
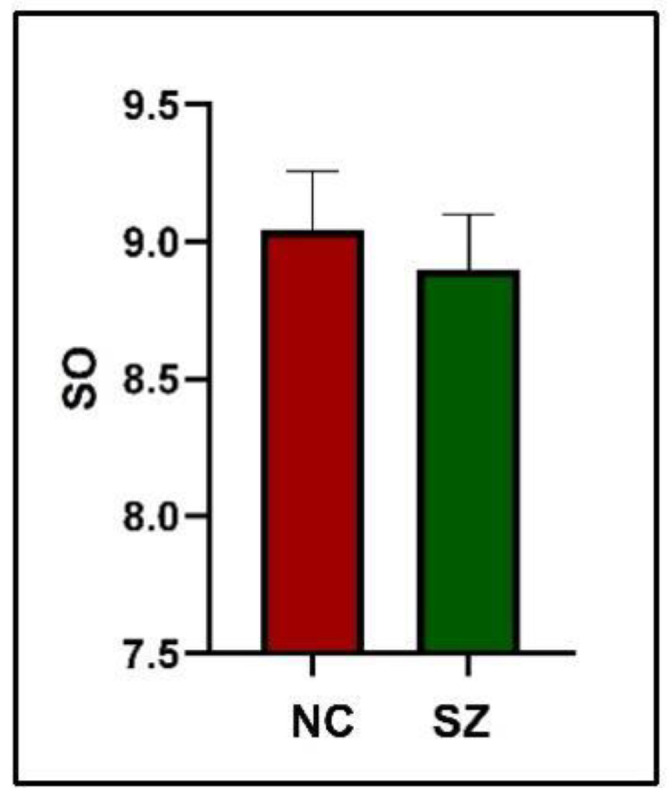
Group differences in spatial overlap. The number (percentage) of spatial co-occurrences for all pairwise comparisons are shown.

In this part of the study, we averaged the spatial overlap for each subject at all time points. As shown in Figure 5, after statistical analysis, the spatial overlap was lower in SZ patients than in NC (p > 0.05).

**Figure 6:**
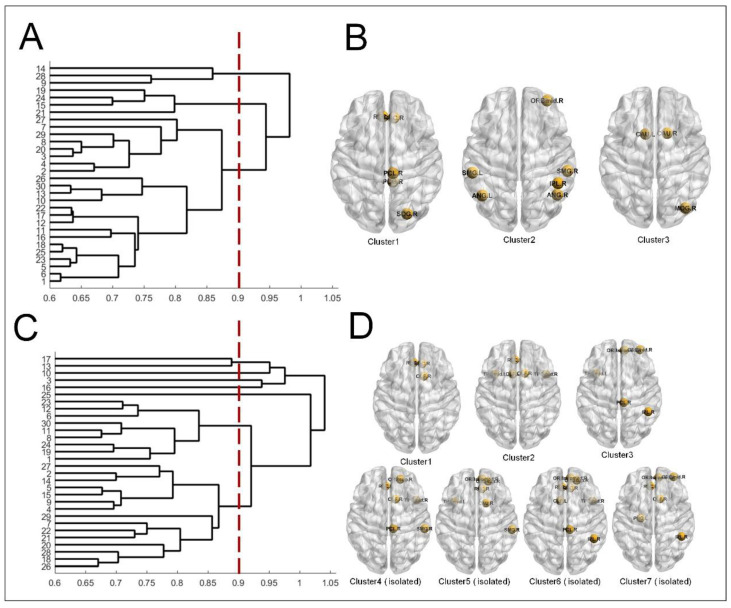
Hierarchical clustering based on the spatial pattern of active hubs: (**A**) NC, (**C**) SZ. Clusters resulting from hierarchical clustering: (**B**) NC (**D**) SZ.

Furthermore, seven clusters emerged in SZ patients after choosing the same threshold of 0.90 for the hierarchical clustering of brain space patterns. That is, the spatial patterns of the brain in SZ patients varied periodically between these seven clusters. However, NC was grouped into only three clusters and varied periodically between the three patterns. We found that the hierarchical clustering patterns of SZ patients were more dispersed (Figure 6C). In contrast, the quasi-cyclic patterns of NC were more stable (Figure 6A). The spatial patterns following the hierarchical clustering of NC are shown in Figure 6B. The spatial patterns following the hierarchical clustering of SZ patients are shown in Figure 6D. Notably, the SZ clustering contained seven spatial patterns, four of which were isolated.

### 3.5. Temporal Overlap of Active Hubs

**Figure 7:**
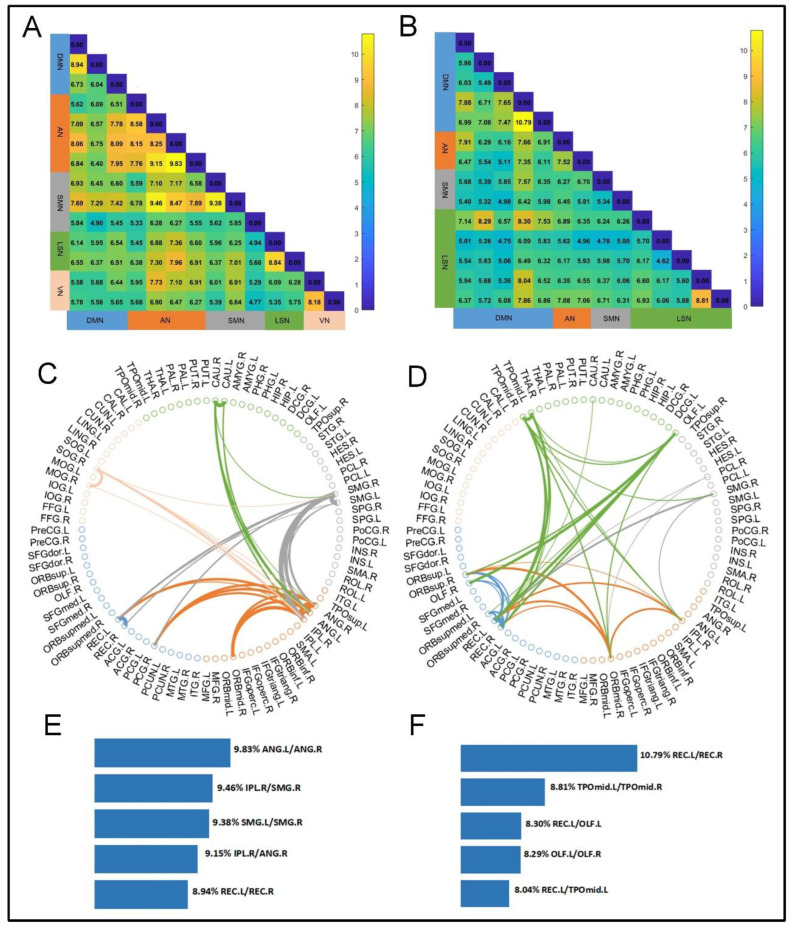
Time overlap of active hubs in the two groups of subjects (expressed as a percentage). (**A**) NC. (**B**) SZ. Distribution in the right and left brain of large temporal overlap in the top 20%. (**C**) NC. (**D**) SZ. The first five significant larger hubs pairs in both groups of subjects. (**E**) NC. (**F**) SZ.

To measure the temporal overlap of active hubs, we calculated the number of points in time that were jointly active on each pair of hubs as a proportion of the total points in time. The results of the temporal overlap are shown in Figure 7. Figure 7A shows the results of the temporal overlap of all hubs in NC. Figure 7B shows the results for SZ patients. Notably, SZ patients had the highest degree of co-occurrence of the REC.L and REC.R located in the DMN, and were also relatively high for the TPOmid.L and TPOmid.R located in LSN, with the remaining significant node pairs being between DMN and LSN. The highest degree of temporal overlap in NC occurred in ANG.L and ANG.R in AN, followed by IPL.R in AN with SMG.R in SMN, with other sizeable temporal overlap pairs in SMN or between SMN and AN. Figure 7C,D shows the distribution of the higher temporal overlap in nodal pairs for NC versus SZ patients. The thickness of the connecting lines in the graph represents the degree of time overlap. The top five most significant temporal overlap hub pairs for NC versus SZ patients are shown in Figure 7E,F.

## 4. Discussion

We used Jackknife Correlation to construct time-varying dynamic brain networks to investigate the spatial and temporal organization patterns of SZ brain hubs. This paper utilizes the BC method in graph theory to identify brain hubs. Meanwhile, the subnetwork distribution is presented, and the hubs are studied from the perspective of the subnetwork distribution. Further, the difference between SZ and NC is analyzed using temporal overlap, spatial overlap, and hierarchical clustering of hubs. The results showed a trend towards decreased repetition and periodicity in the spatial configuration of brain hub activity and abnormalities in hub pair participation preferences in SZ patients. Research on brain hubs based on fMRI studies can help identify abnormal brain activity or connectivity patterns associated with neurological and psychiatric disorders. Comparing brain activity in healthy individuals and those with disorders such as schizophrenia can help elucidate the neural basis of these disorders, potentially aiding early diagnosis, treatment and therapeutic intervention.

### 4.1. Decreased Ability of SZ Patients to Integrate and Process Information

BC represents the ability of a region to integrate information from other brain networks. Our study showed that BC was significantly reduced at the whole-brain level in patients with SZ (Figure 2A). This result suggests that the brain of SZ patients has a reduced ability to integrate and process information. A possible explanation for our findings could be a reduction in the patient’s brain’s global communication capacity. A study exploring the role in the global functioning of brain function in schizophrenia showed that the rich club organization between the hubs was significantly affected in SZ patients. Selective disruption of brain connectivity between the hubs in the patient’s brain may lead to reduced communication capacity and altered functional brain dynamics [32]. Furthermore, a study based on the dynamic reconfiguration of resting-state functional connectivity suggests a fundamental role for elevated informational communication instability and diminished whole-brain integration of higher-order networks in schizophrenia [33]. These correlational studies with our results provide neurological evidence to support the hypothesis of reduced global communication capacity in schizophrenia.

We have also made a set of comparisons of BC from the RSN level. The results showed significant differences in DMN, VN, and AN between SZ patients and NC (Figure 2B). Firstly, for DMN, our results found significantly higher BC in SZ patients than in NC, suggesting the most significant contribution of this network to the compensatory mechanisms caused by the patients’ disrupted information integration [34]. Studies have found that the DMN in SZ patients is frequently over-activated and over-connected [35], which may be associated with excessive self-reference and impairment in attention and working memory [36]. Based on these findings, our results suggest that abnormal DMN states affect the SZ patient’s cognitive function, leading to mental deficits. Secondly, for AN, our results found that SZ patients had significantly lower BC than NC, suggesting a prominent contribution of abnormalities in this network to reducing patients’ global information processing capacity. Abnormalities in attentional networks are closely associated with attentional deficits in schizophrenia [37,38]. Brain dysfunction resulting from abnormalities in the attentional network may lead to abnormal clinical and cognitive measures [39]. Finally, our results found significantly lower BC for VN in SZ patients. This finding further emphasizes the role of impaired visual processing in SZ. Alterations in the VN may contribute prominently to the observed deficits in information processing capacity in this patient population. Evidence suggests that schizophrenia is associated with deficits in higher-order visual information processing [40,41,42]. These findings suggest that abnormal visual network states are associated with SZ dysfunction. Furthermore, our conjecture is confirmed based on the results of the subnetwork distribution (Figure 4). Specifically, we observed a significantly higher proportion of subnetwork distribution in the DMN and a significantly lower proportion of active hubs in the AN and VN in SZ patients compared to NC, confirming the impairment of the AN and VN and the overactivation of the DMN in SZ.

In addition to the between-group differences in BC values that we observed at the whole-brain and subnetwork levels, there were also significant differences between the two groups in terms of candidate hubs (Figure 3). Specifically, when examining the inconsistent structure of candidate hubs in the SZ versus NC, we found that inconsistent candidate hubs in SZ patients were located in the DMN and LSN, potentially reflecting altered functional connectivity in these regions associated with SZ. These findings are consistent with previous studies that SZ is associated with regional differences in individual nodes of the DMN [43] and that hubs of SZ are more common in the LSN [44]. Our findings highlight differences in hub patterns between the SZ and NC and suggest that these differences may underlie observed differences in information processing capacity and cognitive function between the two groups.

### 4.2. Reduced Stability of Spatial Configuration of Brain Activity in SZ Patients

Recent research has found that the activities of the cerebral cortex are not separate. Instead, they overlap and interact at different scales, which may be attributable to dynamic fluctuations in human brain activity in space and time [45], and is associated with heterogeneous activity of mixed neurons within brain regions. Evidence from the study suggests that network overlap in the brain may provide clues to help understand network interactions in human cognition [46]. Describing the spatiotemporal organization of the brain and the differences between SZ patients and NC is also one of the central questions in the study of SZ as a psychiatric disorder [47]. We therefore combine this concept of overlap with spatiotemporal dynamics to investigate the dynamic interaction of abnormal spatiotemporal patterns in the brain hubs of SZ patients. Research reveals abnormal spatiotemporal patterns that indicate disrupted communication and coordination between brain regions in schizophrenia patients [48,49]. Understanding these spatiotemporal changes is critical to uncovering the neural mechanisms of schizophrenia and developing more targeted treatments or interventions [50,51].

Our study found that, on average, patients with SZ had lower spatial overlap than NC. Further, after hierarchical clustering based on the spatial overlap, we obtained the clustering patterns of quasi-periodic changes in the two groups of subjects. A quasi-periodic pattern is a recurring pattern of brain activity [52]. The clustering patterns were found to be more dispersed in the SZ patients, indicating an abnormal quasi-periodic pattern in the SZ patients [53]. These disordered quasi-periodic patterns may lead to abnormal functional connectivity in patients with SZ. This conjecture is supported by other psychiatric disorders, where disruption of the patient’s quasi-periodic patterns may result in a failure of the quasi-periodic patterns to maintain normal functional connectivity [54]. In contrast to NC’s spatial patterns after clustering, the altered hubs in SZ’s clustering patterns contain hubs such as TPOmid.L and TPOmid.R nodes. The bilateral TPOmid nodes are located in the temporal poles, the part of the temporal lobe that forms the most rostral part of the temporal lobe. The temporal pole is associated with higher cognitive processes and symptoms associated with temporal pole lesions or dysfunction can help identify schizophrenia [55]. In examining dynamic functional connectivity disorders associated with schizophrenia, Sun and colleagues found that patients with SZ had abnormal temporal area properties of the TPOmid bilaterally [56]. However, in contrast to NC, no ANG.R and ANG.L nodes located in AN were found in the post-clustering spatial patterns of the SZ patients. The bilateral ANG nodes are located in the posterior part of the lower parietal lobe and play a role in semantic processing, reading, and word comprehension [57]. The memory loss and slower cognitive speed exhibited by SZ patients may be related to the loss of ANG in their patterns. A study by Gao and colleagues also found that people with SZ showed reduced low-frequency fluctuations in the ANG [58]. Thus, there were abnormalities in the spatial patterns of SZ patients, and the changes in spatial patterns were more variable in SZ patients. These findings support the theory of spatial dynamics, whereby the spatial patterns of the brain change continuously over time. Furthermore, the spatial patterns of clustering reflect periodic changes in the spatial patterns of the brain. Research has shown a correlation between quasi-periodic patterns in the brain activity of individuals and global signals [52,59]. Global signals are common patterns of neural activity observed throughout the brain. The widespread abnormal global signals found in the SZ may lead to alterations in the SZ quasi-periodic pattern [60]. Here, we investigate the dynamics of brain abnormalities in SZ patients by examining quasi-periodic patterns in the brain. The quasi-periodic patterns provide insight into the mechanisms behind the differences in spatial patterns in SZ patients.

### 4.3. Abnormal Pair Participation Preference for Hubs in SZ Patients

Hub nodes play a key role in the brain’s functional brain connectivity network. They are important nodes that connect different brain regions and facilitate the dissemination and integration of information in the brain network. The activity of these hub nodes may increase when the brain has to process a large flow of information, creating a pattern of temporary, paired occurrences. These temporary hub connections provide a faster, more direct pathway to meet the demands of neuronal traffic. These temporary fast connections may facilitate the rapid transfer and processing of information. We use temporal overlap as a measure of the strength of such fast connections. Time overlap refers to the percentage of time that two hubs are active simultaneously. The temporal overlap reflects the temporal interaction pattern between hubs and indicates pair participation preference for hubs [61]. Overall, the temporal overlap was lower in SZ patients than in NC, suggesting that SZ patients have a reduced ability to interact with information. Our study shows that the hub pairs with the strongest participation preference in SZ patients are distributed between REC.L and REC.R. REC is the anterior frontal cortex of the anterior cingulate, which is implicated in the psychopathology of schizophrenia [62]. These regions are associated with olfactory function. A large proportion of SZ patients have olfactory hallucinations [63,64] and therefore prolonged abnormal activation of these regions may be associated with these olfactory abnormalities. Unusually, hubs’ highest pair participation preference was found between ANG.L and ANG.R in NC. Located in the posterior part of the brain [65], the ANG is part of the DMN and is involved in self-referential processing, imagery, and memory, playing an important role in language processes, spatial cognition, and memory retrieval [66]. The findings suggest that a control mechanism involving the ANG contributes to perceptual and cognitive problems in patients with SZ, which is essentially a neural communication problem, and that this control mechanism may be a worthy target for intervention [67]. Connectivity analysis has shown that abnormal information transfer between ANG areas in patients with SZ may be due to reduced connectivity between the ANG and frontal action-generating areas [68]. More specifically, ANG has become a cross-modal hub in which fused multisensory information is combined and integrated to allow it to make sense of events in a comprehensive and meaningful way and to redirect attention to relevant information. Furthermore, the dysfunction of ANG has been proposed in neurobiological models of social deficits and ego disorders observed in SZ patients [69]. In NC, brain nodes located in the ANG were active and were consistently active. Therefore, patients with SZ may have cognitive dysfunction due to abnormal pair participation preference for hubs in the ANG nodes. Our analysis also revealed that SZ patients showed an abnormal reduction in pair participation preference for SMG.L and SMG.R compared to NC. These SMG nodes are located in the parietal lobe, above the temporal lobe, in the vicinity of brain regions involved in language and hearing [70]. As a branch of the inferior parietal lobule, the SMG plays an important role in orientation regulation, proprioception, and emotion regulation [71], and this region has been repeatedly identified as abnormal in structural imaging studies of SZ patients [72]. SMG nodes play a crucial role in the processing of heard speech, and their abnormally reduced pattern of paired participation may contribute to the language-related deficits observed in SZ patients. These changes in pair participation preferences between hubs in the SZ represent a significant change in the rapid pathway of information transfer and processing of hubs. 

## 5. Limitations and Directions for Future Research

This study had some limitations, mainly due to the small sample size. Although we obtained relatively stable and reliable results, the universality of the sample still needs to be explored and improved by recruiting more patients in future surveys. The study adopted a cross-sectional design, offering a snapshot of brain dynamics at a specific moment in time in individuals diagnosed with schizophrenia. However, to comprehensively grasp the dynamic nature of schizophrenia, future investigations employing longitudinal studies are imperative. Furthermore, in order to clarify the correlation and causality in the differences in brain network organization between patients with schizophrenia and healthy controls, future research directions should use longitudinal studies to track brain changes before and after the onset of illness.

## 6. Conclusions

This study employed a time-varying network model to investigate the spatial and temporal overlap of brain hubs in SZ patients. The findings revealed a notable decrease in Betweenness Centrality observed across multiple levels in SZ patients. Our results contribute substantiating evidence indicating that hubs in both healthy individuals (NC) and SZ patients undergo dynamic fluctuations even during resting states. However, hubs in SZ individuals demonstrate heightened spatial and temporal instability in their organization, manifesting frequent spatial reconfigurations and deviant pairwise engagement preferences. The identification of aberrant brain regions in this study offers promising avenues for further in-depth pathological investigations into SZ.

## Figures and Tables

**Table 1:** Demographic and clinical characteristics of the participants in this study.

Characteristic	SZ	NC	Statistical Test
Number of subjects	43	49	--
Age (years)	34.84 ± 8.60	33.12 ± 8.22	*p* = 0.331
Sex (male/female) SAPS	30/13 30.61 ± 20.26	30/19 --	*p* = 0.112 --

Note: The values are denoted as mean ± standard deviation.

## Data Availability

Data are contained within the article.

## Appendix A

**Table A1:** Mapping between ROI names and experiential function networks in AAL template.

ROI	Regions	Abbreviated	RSN
1	Precentral_L	PreCG.L	DMN
2	Precentral_R	PreCG.R	DMN
3	Frontal_Sup_L	SFGdor.L	DMN
4	Frontal_Sup_R	SFGdor.R	DMN
5	Frontal_Sup_Orb_L	ORBsup.L	DMN
6	Frontal_Sup_Orb_R	ORBsup.R	DMN
7	Frontal_Mid_L	MFG.L	Attention
8	Frontal_Mid_R	MFG.R	Attention
9	Frontal_Mid_Orb_L	ORBmid.L	Attention
10	Frontal_Mid_Orb_R	ORBmid.R	Attention
11	Frontal_Inf_Oper_L	IFGoperc.L	Attention
12	Frontal_Inf_Oper_R	IFGoperc.R	Attention
13	Frontal_Inf_Tri_L	IFGtriang.L	Attention
14	Frontal_Inf_Tri_R	IFGtriang.R	Attention
15	Frontal_Inf_Orb_L	ORBinf.L	Attention
16	Frontal_Inf_Orb_R	ORBinf.R	Attention
17	Rolandic_Oper_L	ROL.L	Sensorimotor
18	Rolandic_Oper_R	ROL.R	Sensorimotor
19	Supp_Motor_Area_L	SMA.L	Attention
20	Supp_Motor_Area_R	SMA.R	Sensorimotor
21	Olfactory_L	OLF.L	Subcortical
22	Olfactory_R	OLF.R	DMN
23	Frontal_Sup_Medial_L	SFGmed.L	DMN
24	Frontal_Sup_Medial_R	SFGmed.R	DMN
25	Frontal_Mid_Orb_L	ORBsupmed.L	DMN
26	Frontal_Mid_Orb_R	ORBsupmed.R	DMN
27	Rectus_L	REC.L	DMN
28	Rectus_R	REC.R	DMN
29	Insula_L	INS.L	Sensorimotor
30	Insula_R	INS.R	Sensorimotor
31	Cingulum_Ant_L	ACG.L	DMN
32	Cingulum_Ant_R	ACG.R	DMN
33	Cingulum_Mid_L	DCG.L	Subcortical
34	Cingulum_Mid_R	DCG.R	Subcortical
35	Cingulum_Post_L	PCG.L	DMN
36	Cingulum_Post_R	PCG.R	DMN
37	Hippocampus_L	HIP.L	Subcortical
38	Hippocampus_R	HIP.R	Subcortical
39	ParaHippocampal_L	PHG.L	Subcortical
40	ParaHippocampal_R	PHG.R	Subcortical
41	Amygdala_L	AMYG.L	Subcortical
42	Amygdala_R	AMYG.R	Subcortical
43	Calcarine_L	CAL.L	Visual
44	Calcarine_R	CAL.R	Visual
45	Cuneus_L	CUN.L	Visual
46	Cuneus_R	CUN.R	Visual
47	Lingual_L	LING.L	Visual
48	Lingual_R	LING.R	Visual
49	Occipital_Sup_L	SOG.L	Visual
50	Occipital_Sup_R	SOG.R	Visual
51	Occipital_Mid_L	MOG.L	Visual
52	Occipital_Mid_R	MOG.R	Visual
53	Occipital_Inf_L	IOG.L	Visual
54	Occipital_Inf_R	IOG.R	Visual
55	Fusiform_L	FFG.L	Visual
56	Fusiform_R	FFG.R	Visual
57	Postcentral_L	PoCG.L	Sensorimotor
58	Postcentral_R	PoCG.R	Sensorimotor
59	Parietal_Sup_L	SPG.L	Sensorimotor
60	Parietal_Sup_R	SPG.R	Sensorimotor
61	Parietal_Inf_L	IPL.L	Attention
62	Parietal_Inf_R	IPL.R	Attention
63	SupraMarginal_L	SMG.L	Sensorimotor
64	SupraMarginal_R	SMG.R	Sensorimotor
65	Angular_L	ANG.L	Attention
66	Angular_R	ANG.R	Attention
67	Precuneus_L	PCUN.L	DMN
68	Precuneus_R	PCUN.R	DMN
69	Paracentral_Lobule_L	PCL.L	Sensorimotor
70	Paracentral_Lobule_R	PCL.R	Sensorimotor
71	Caudate_L	CAU.L	Subcortical
72	Caudate_R	CAU.R	Subcortical
73	Putamen_L	PUT.L	Subcortical
74	Putamen_R	PUT.R	Subcortical
75	Pallidum_L	PAL.L	Subcortical
76	Pallidum_R	PAL.R	Subcortical
77	Thalamus_L	THA.L	Subcortical
78	Thalamus_R	THA.R	Subcortical
79	Heschl_L	HES.L	Sensorimotor
80	Heschl_R	HES.R	Sensorimotor
81	Temporal_Sup_L	STG.L	Sensorimotor
82	Temporal_Sup_R	STG.R	Sensorimotor
83	Temporal_Pole_Sup_L	TPOsup.L	Attention
84	Temporal_Pole_Sup_R	TPOsup.R	Sensorimotor
85	Temporal_Mid_L	MTG.L	DMN
86	Temporal_Mid_R	MTG.R	DMN
87	Temporal_Pole_Mid_L	TPOmid.L	Subcortical
88	Temporal_Pole_Mid_R	TPOmid.R	Subcortical
89	Temporal_Inf_L	ITG.L	Attention
90	Temporal_Inf_R	ITG.R	DMN
